# [Corrigendum] Epithelial-mesenchymal transition is necessary for acquired resistance to cisplatin and increases the metastatic potential of nasopharyngeal carcinoma cells

**DOI:** 10.3892/ijmm.2025.5591

**Published:** 2025-07-21

**Authors:** Pei Zhang, Hao Liu, Fei Xia, Qian Wen Zhang, Yuan Yuan Zhang, Qing Zhao, Zhen Hua Chao, Zhi Wen Jiang, Chen Chen Jiang

Int J Mol Med 33: 151-159, 2014; DOI: 10.3892/ijmm.2013.1538

Following the publication of the above article, an interested reader drew to the authors' attention that Figs. 3B and 4B contained western blot data (specifically, the MMP-9 and Vimentin western blots in Figs. 3B and 4B, respectively) that were more similar than expected. Moreover, further strikingly similar data were also identified examining the western blot data within Fig. 4B and 4D, and also comparing the western blots in Figs. 1D, 3B, 4B and 4D with data featured in an article in the journal *Oncotarget* that was published subsequently to the above article, but which featured the first author (Pei Zhang) as an author in common.

The authors were able to re-examine their original data (which were also presented to the Editorial Office), and confirmed that the Vimentin data in Fig. 4B and the ZEB1 data in Fig. 4D were the same western bands as those featured for the MMP-9 data in Fig. 3B and the Fibronectin data in Fig. 4B, respectively. These errors were made during the assembly of Fig. 4 (the data for MMP-9 and Fibronectin were inadvertently duplicated in the figure). The corrected version of Fig. 4, now displaying the accurate experimental data for the Vimentin (Fig. 4B) and ZEB1 (Fig. 4D) blots in the CNE2Z and CNE2Z/DDP groups, is shown on the next page. Furthermore, regarding the issue of image duplication comparing between the above article published in 2014 and the one published in *Oncotarget* in 2015, the authors will contact the *Oncotarget* journal to handle the necessary corrections.

The authors can confirm that the errors associated with this figure did not have any significant impact on either the results or the conclusions reported in this study, and all the authors agree with the publication of this Corrigendum. The authors are grateful to the Editor of *International Journal of Molecular Medicine* for allowing them the opportunity to publish this Corrigendum; furthermore, they apologize to the readership of the Journal for any inconvenience caused.

## Figures and Tables

**Figure 4:**
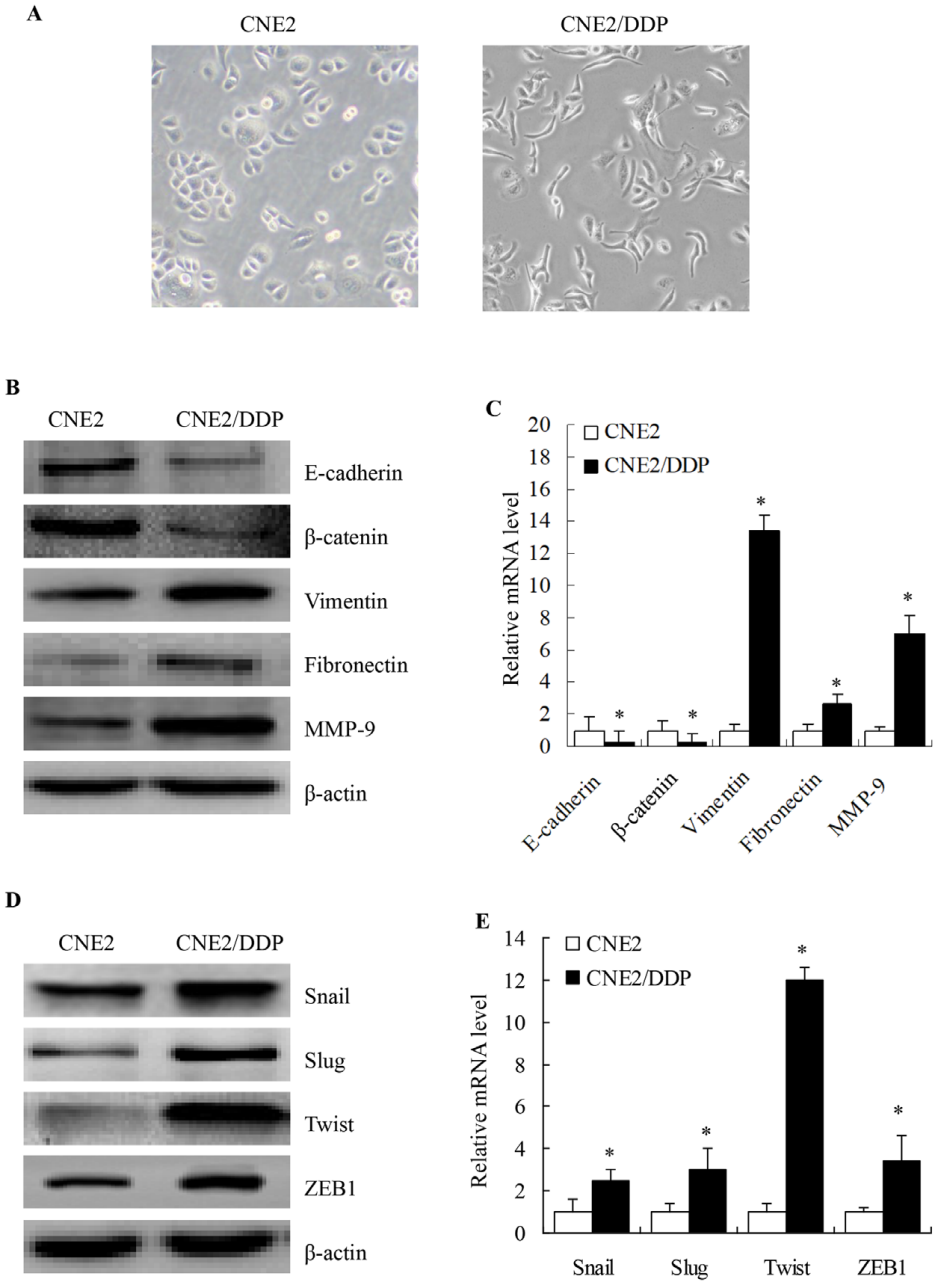
Cisplatin (DDP)-treated CNE2 cells underwent an epithelial-mesenchymal transition (EMT)-like transformation. (A) Cell morphology was observed by microscopy at x200 magnification. Parental CNE2 cells showed an epithelioid, cobblestone appearance, similar to HNE1 cells. By contrast, the morphology of CNE2/DDP cells was of mixed type, showing an epithelioid, long, spindle/fibroblastic pattern with pseudopodia and an unorganized growth pattern. (B) Expression levels of the EMT-related proteins, E-cadherin, β-catenin, vimentin, fibronectin and matrix metalloproteinase (MMP)-9, were determined by western blot analysis. The downregulation of E-cadherin and β-catenin and upregulation of vimentin, fibronectin and MMP-9 was observed in CNE2/DDP cells. (C) mRNA expression levels of E-cadherin, β-catenin, vimentin, fibronectin and MMP-9 genes were analyzed by qRT-PCR (means ± SEM, n=3, ^*^P<0.05 compared with the parental CNE2 cells). (D) Increased protein levels of the EMT-related transcription factors, Snail, Slug, Twist and zinc finger E-box binding homeobox 1 (ZEB1), were determined by western blot analysis. (E) mRNA expression levels for genes coding for the EMT-related transcription factors, Snail, Slug, Twist and ZEB1, were analyzed by qRT-PCR (means ± SEM, n=3, ^*^P<0.05 compared with parental CNE2 cells).

